# Supplementary material for: Six-fold increase of atmospheric pCO2 during the Permian–Triassic mass extinction
Source: Nat Commun. 2021 Apr 9;12:2137. doi: 10.1038/s41467-021-22298-7 (PMC8035180; doi:10.1038/s41467-021-22298-7)
Supplement: Supplementary file 1 — Supplementary Information [file 41467_2021_22298_MOESM1_ESM.pdf]

Supplementary Information

Six-fold increase of atmospheric pCO_2 during the Permian–Triassic mass extinction

Yuyang Wu[1, 2], Daoliang Chu[1*], Jinnan Tong[1], Haijun Song[1], Jacopo Dal Corso[1], Paul B. Wignall[3], Huyue Song[1], Yong Du[1], Ying Cui[2*]

[1]State Key Laboratory of Biogeology and Environmental Geology, School of Earth Sciences, China University of Geosciences, Wuhan, China

[2]Department of Earth and Environmental Studies, Montclair State University, Montclair, U.S.A.

[3]School of Earth and Environment, University of Leeds, Leeds, UK

Corresponding authors: chudl@cug.edu.cn; cuiy@montclair.edu

Supplementary Note 1. Geological setting

The study area is located in the western Guizhou and eastern Yunnan provinces of southwestern China that were in the east of Palaeotethys, in tropical latitudes during the Late Permian to Early Triassic (Supplementary Fig. 1). The terrestrial and marginal marine siliciclastic facies are composed of the Xuanwei Formation, Kayitou Formation and Dongchuan Formation in ascending order (Supplementary Fig. 2). The Xuanwei Formation consists of greenish sandstone and grey-greenish to dark grey mudstone intercalated with abundant coal beds. The Kayitou Formation is characteristic by grey-greenish silty mudstone and mudstone but no coal beds. The Grey-greenish mudstone change to yellow-green and yellow in some weathered outcrops. The Dongchuan Formation comprises purple-red sandstones and siltstones interbedded with mudstones. The sedimentary environment and lithology of the Kayitou and Dongchuan formations are spatially heterogeneous across southwestern China (Supplementary Fig. 1).

In total, four study sections are involved: Chahe, Jiucaichong, ZK4703 core and Chinahe. The Chahe and Jiucaichong sections are located in the western part of the study area are more proximal to the hinterland, and their shallow lake/floodplain facies yield fossils plants, conchostracans and insects[1, 2]. The Late Permian peatland *Gigantopteris* flora began to decreases in the uppermost part of Xuanwei Formation and completely vanished at 1 m (Chahe) and 0.7 m (Jiucaichong) above the last coal bed in the Kayitou Formation (i.e. position of last appearance datum (LAD) coal). The flora was replaced by *Peltaspermum* in the overlying strata, accompanied by the conchostracan *Euestheria gutta*[1]. The Chinahe section, and ZK4703 core are located more distally and record brackish, lagoonal sediments (Supplementary Fig. 1) yielding a mixed continental-marine biota, including marine bivalves, lingulid brachiopods, terrestrial plants, conchostracans and insects[1, 3]. The Xuanwei Formation contains abundant Late Permian fossil plants in Chinahe, especially the high diversity, abundant *Gigantopteris* flora. The species of *Gigantopteris* flora in Chinahe began to decrease at the top of Xuanwei Formation and completely vanished at ~1 m above the top of Xuanwei Formation. The extinction of *Gigantopteris* flora and fossil plant could also be observed in ZK4703 core by the plant fragments. In both records the flora was replaced by a low diversity and less abundant *Annalepis* (lycopsid) and *Peltaspermum* (seed ferns) dominated flora. Meanwhile, the *Euestheria gutta*-bearing conchostracan fauna and marine bivalves and lingulid brachiopods are observed after the extinction of *Gigantopteris* flora in Kayitou Formation[1, 4].

Supplementary Note 2. Results of organic carbon isotopes and total organic carbon

The $\delta^{13}C_{bulk}$ and $\delta^{13}C_{p}$ ($\delta^{13}C_{cuticle}$ and $\delta^{13}C_{wood}$) profiles of the four study sections exhibit nearly the same secular trends. Each profile can be divided into four stages: (1) a pre-CIE stage, (2) an onset of the negative CIE (onset of CIE) stage, (3) a prolonged CIE body stage and (4) a post-CIE stage. For the studied sections, these four stages roughly correspond to the Xuanwei Formation, base of Kayitou Formation, almost whole Kayitou Formation and uppermost Kayitou to Dongchuan Formation.

At Chinahe, the mean values of $\delta^{13}C_{bulk}$, $\delta^{13}C_{cuticle}$ and $\delta^{13}C_{wood}$ at pre-CIE stage are –25.2‰, –24.7‰ and –24‰ respectively. Then a prominent and gradually drop occurred at the base of the Kayitou Formation with a sharp spike of –30.8‰, –31.9‰ and –31.9‰ in bulk organic matter, cuticle and wood (including charred wood and non-charred wood), which is defined as the onset of the CIE. This is followed by a CIE body with prolonged low values (< –30‰) through the whole Kayitou Formation, but interrupted by a slight positive shift within the lower part of the Formation. In the post-CIE stage, a recovery to higher $\delta^{13}C_{bulk}$, $\delta^{13}C_{cuticle}$ and $\delta^{13}C_{wood}$ values that average –27.3‰, 29.2‰ and 28.9‰ respectively commences in the uppermost part of the Kayitou Formation.

The Xuanwei Formation in the ZK4703 core is characterized by $\delta^{13}C_{bulk}$, $\delta^{13}C_{cuticle}$ and $\delta^{13}C_{wood}$ values that average –24.6‰, –25.4‰, –24.3‰ in the pre-CIE stage. Then $\delta^{13}C_{bulk}$, $\delta^{13}C_{cuticle}$ and $\delta^{13}C_{wood}$ values drop gradually to first peak values of –30.7‰, –32.6‰ and –30.8‰ at the onset of the CIE stage. In the CIE body stage, the three carbon isotope profiles are characterized by sustained low values (< –30‰), interrupted by a slight positive shift within lower Kayitou Formation. The recovery to higher values up to –27.6‰ is recorded in bulk organic matter.

In the Jiucaichong section, the mean values of $\delta^{13}C_{bulk}$, $\delta^{13}C_{cuticle}$ and $\delta^{13}C_{wood}$ in the pre-CIE stage are –25.4‰, –25‰ and –23.6‰ respectively, but they are interrupted by several more negative values in the uppermost part of the Xuanwei Formation. The onset of the CIE is observed at the base of the Kayitou Formation, it shows lowest values up to –29.8‰ and –30.2‰ in bulk organic matter and plant cuticle. The CIE body stage is also interrupted by a slight positive shift. In the post-CIE stage, the $\delta^{13}C_{bulk}$ and $\delta^{13}C_{cuticle}$ values average –27.1‰ and –27.8‰ respectively.

At Chahe section, the $\delta^{13}C_{bulk}$ data[5, 6] and $\delta^{13}C_{wood}$ values in the pre-CIE stage are around –24.6‰ and –24.0‰ respectively throughout the Xuanwei basal Kayitou formations, and subsequently drop gradually to first peak values of –29.2‰ and –29.4‰ at the onset of the CIE

stage. The prolonged and low values (ca. –29‰) persist in the Kayitou Formation. The recovery to higher values up to –27‰ is recorded in bulk organic matter.

The $\delta^{13}C_{cuticle}$ values are usually lighter than $\delta^{13}C_{wood}$ values by about 1~2‰ from the three study sections except in the CIE body stage at Chinahe and Jiucaichong. Smaller $\delta^{13}C$ values recorded in C_3 plant cuticle compared with bulk organic matter could be observed in most stages, although there are no significant differences in the pre-CIE and onset of CIE stages from Chinahe and Jiucaichong. The difference between $\delta^{13}C_{wood}$ values and $\delta^{13}C_{bulk}$ vary significantly among different stages and sections. For example, $\delta^{13}C_{wood}$ values at pre-CIE are higher than $\delta^{13}C_{bulk}$ values from Chinahe, whereas higher $\delta^{13}C_{bulk}$ values are observed in the CIE body stage.

Total organic carbon (TOC) concentrations in the pre-CIE and onset of CIE stage range from 0.4% to 2.9% at Chinahe section, and from 0.2% to 3.2% in the ZK4703 core. This is followed by a pronounced decrease of TOC in the CIE body and post-CIE stage, where it ranges from 0.1% to 0.4% at Chinahe, from 0.2% to 0.8% at ZK4703. At Jiucaichong, the decrease of TOC happened at the base of the onset of the CIE stage. TOC concentrations range from 0.03% to 1.9% at pre-CIE and the base of onset of CIE stage, followed by a pronounced decrease of TOC (0.02% to 0.25%) in the overlying strata.

Supplementary Note 3. Facies association description

The lithofacies around the formation boundary was examined in detail at both sections and found to be comparable. The Chinahe section provides the best record because it is unaffected by faulting (Supplementary Fig. 8). Five lithofacies occur in the boundary interval:

i. Coal. The two highest coals seams of the Xuanwei Formation were examined; the lower example rests on a palaeosol consisting of rootlets in a pale grey clay, the upper example rests on a thin carbonaceous shale rich in plant debris.

ii. Thinly-bedded mudstone and siltstone. Beds range from 20 – 70 cm and grain size can

reach very fine sand. Bed thickness is typically 2 – 3 mm and bedding surfaces are often covered in charcoal and plant debris. The mudstone contains grains of chamosite and kaolinite which are interpreted to be authigenic, but reworked and transported in the siltstone[2].

iii. Massive, green sandstone. Beds are sharp, sometimes erosive based, and range from 20-75 cm thick. Grain size is medium to coarse sand and bed tops can show rapid fining. There are no internal structures and organic content is high (plant debris and cm-sized charcoal chunks), whilst chamosite is the dominant grain type.

iv. Grey-green mudstone. Only present in the Kayitou Formation, the mudstone often shows thin (<1 mm) laminae that are widely spaced (>1 cm). Small, tabular carbonate concretions are common and a conchostracan (*Euestheria gutta*) and plants of Triassic affinity (*Annalepis*, *Peltaspermum*) are seen. This lithofacies is the only one to have a pyrite content, of crystals and framboids[2].

v. Interbedded mudstone and sandstone. The uppermost part of the logged section sees the arrival of thin beds of sandstone that increase in thickness (from ~ 1 mm to 2cm) and abundance upwards over 2 m of section.

Interpretation: The Chinahe location records low energy coastal swamp conditions during the Permian-Triassic boundary interval. Absence of wave processes points to a sheltered setting, either behind protective islands or on a broad, gentle shelf in which basinal processes were dampened. Although low energy, fine-grained deposition dominated depositional history, the introduction of sand either occurred in regular, minor influxes (facies ii) or as major high-energy events that saw the rapid deposition of coarse sand composed of material reworked (chamosite, coal detritus) from the paralic swamps and lakes (lithofacies i and ii). Such event beds indicate major flood events regularly swept through the coastal environments and suggest a flashy discharge regime in the coastal rivers[2]. No significant change in depositional style was noted between the Xuanwei and Kayitou formations, other than the loss of coals due to the plant extinction, and the brief appearance of marine-influenced (i.e. pyritic) lithofacies due a basal Triassic transgression[2]. The subsequent progradation saw the

reappearance of the same lithofacies: reworked, green chamositic sandstones in paralic mudstones indicating no significant precipitation or climate change in the study interval.

Supplementary Note 4. Age model

The age model of study sections is based on an argument that terrestrial $\delta^{13}C_{org}$ and global marine $\delta^{13}C_{carb}$ profiles exhibit isochronous or nearly isochronous long term variances, and is supported by floral, bivalve and conchostracan biostratigraphy (Supplementary Fig. 6; refs[1,4]). Biostratigraphy evidence indicates that the estimated PTB (e-PTB) is in the lower Kayitou Formation slightly above the onset of the CIE. Similarly, within marine PTB sections the PTB is immediately above the onset of CIE seen in global marine $\delta^{13}C_{carb}$ values. Previous studies have placed the e-PTB significantly higher, in the uppermost part of the Kayitou Formation or even the basal part of the Dongchuan Formation based on U-Pb age dates[5, 6]. Based on biostratigraphic constraints and similar CIE patterns, our study demonstrates a robust correlation of terrestrial $\delta^{13}C_{org}$ profiles with the global marine $\delta^{13}C_{carb}$ records, which implies an isochronous (or nearly isochronous) CIE event.

The detailed age model is calculated from stratigraphic depth and U-Pb dating from GSSP Meishan[7]. The 4.5 m level at Chinahe (688.5 m at ZK4703; 0.5 m at Jiucaichong; 71 m at Chahe) is thought to date to 252.104 Ma before onset of CIE. The strata from 25.05 m at Chinahe (674.75 m at ZK4703; 7.85 m at Jiucaichong; 87.2 m at Chahe) to 29.55 m (671.75 m at ZK4703; 10.95 m at Jiucaichong; 89 m at Chahe), defined as onset of CIE (and corresponding to the onset of CIE in marine $\delta^{13}C_{carb}$) are equivalent to 251.967 Ma and 251.902 Ma respectively. A slight recovery is found at 32.5 m (670.25 m at ZK4703; 14.35 m at Jiucaichong; 94 m at Chahe) at 251.774 Ma. A 251.568 Ma age is assigned to the second peak negative CIE at 48.55 m (664 m at ZK4703; 17.02 m at Jiucaichong). Finally, the 65.95 m level (655 m at ZK4703; 25.5 m at Jiucaichong) is of 251.4 Ma age.

Supplementary Note 5. Conodont biostratigraphy

The conodont zone sequence of the GSSP at Meishan provides the standard to correlate with other sections and includes, in ascending order, the *C. changxingensis* zone, *C. yini* zone, *C. meishanensis* zone, *H. changxingensis* zone, *C. taylorae* zone, *H. parvus* zone, *I. staeschei* zone, and *I. isarcica* zone[8]. In another zonation scheme[9], the *H. changxingensis* and *C. taylorae* zones are equal to the *C. zhejiangensis-H. changxingensis* zone. The *C. changxingensis* zone can be found in most sections. However, there are some issues when correlating overlying conodonts zone from Meishan to other sections, because of hiatuses, regionally-distinct conodont species and low-resolution conodont zones. Shallow water area in eastern Palaeotethys, especially the isolated carbonate platforms in South China, witnessed rapid regression during the latest Permian, which caused sedimentary gaps and loss of some conodont zones from the *C. yini* zone to the *C. taylorae* zone. For example, the *C. meishanensis* zone is absent in the Yangou section[8]. The latest Permian *C. yini* and *C. meishanensis* species are not common in some shallow water area, instead, *H. latidentatus* zone and *H. praeparvus* zone take the place of *C. yini* and *C. meishanensis* in shallow water areas in eastern and western Palaeotethys respectively[10]. Other differences exist between Latest Permian conodonts of Central Palaeotethys, North Neotethys and Eastern Palaeotethys. The endemic species *C. nodosa*, *C. bachmanni*, *C. hauschkei*, and *C. abadehensis* from Central Palaeotethys and North Neotethys have never been found in Eastern Palaeotethys. However, the former two are suggested as regional representatives of *C. yini*, and latter two species are geographic clines of *C. meishanensis*, allowing stratigraphic correlation of these three areas[11, 12, 13]. In addition, the earliest Triassic *H. lobata* zone found in Central Palaeotethys is lumped with the *H. parvus* zone. The *I. staeschei* zone is also lumped with *I. isarcica*.

Supplementary Note 6. Sensitivity Analysis

The error of all the inputs (Supplementary Table 4) affect the uncertainty of $pCO_{2(t)}$. Cui and Schubert[14] performed a sensitivity analysis on the C3 plant proxy and found that the error

of $pCO_{2(t)}$ increases with increasing $pCO2_{(t)}$. We evaluated the effect of uncertainty on three inputs (i.e. $pCO_{2(t=0)}$, $\delta^{13}C_p$ and $\delta^{13}C_{carb}$) on the errors in $pCO_{2(t)}$ compared with full error propagation in this case study (Supplementary Fig. 9). Our sensitivity analysis shows that the error of $pCO_{2(t)}$ increases with increasing $pCO2_{(t)}$ and positive errors are usually larger than negative errors in all scenarios. Full error propagate show positive error reaching ~3000 ppmv and negative error reaching ~900 ppmv at $pCO_{2(t)}$ = 2000 ppmv. The input $pCO_{2(t=0)}$ is set 425±68 ppmv determined from independent stomatal proxies based on fossil conifers from the Dalong Formation in South China. Errors of ±68 ppmv generate positive errors reaching ~1400 ppmv and negative errors reaching ~700 ppmv at $pCO_{2(t)}$ = 2000 ppmv. The input $\delta^{13}C_p$ is set as a mixture of $\delta^{13}C$ in C_3 plant cuticle, charred wood and non-charred wood from southwestern China. The errors of $\delta^{13}C_p$ calculated from LOESS fit are various from 0.20‰ to 0.63‰, which produces positive error reaching ~500 ppmv and negative error reaching ~350 ppmv at $pCO_{2(t)}$ = 2000 ppmv. As the variance in errors of $\delta^{13}C_p$, the relationship between error and pCO_2 is not typically monotone increasing/decreasing. The errors of $\delta^{13}C_{carb}$ calculated from LOESS fit are various from 0.09‰ to 0.14‰, which produces positive error reaching ~150 ppmv and negative error reaching ~150 ppmv at $pCO_{2(t)}$ = 2000 ppmv. Thus, errors of $pCO_{2(t=0)}$ have largest effect on the error of $pCO_{2(t)}$, and the errors in $\delta^{13}C_{carb}$ generate the smallest effect. In addition, the positive error increases rapidly (peak ~6000 ppmv) at $pCO_{2(t)}$ > 2000 ppmv, but negative error remains < 1500 ppmv across the entire range of $pCO_{2(t)}$ in all scenarios.

Supplementary Table 1. Statistical summary and non-parameter tests of marine and terrestrial CIE magnitude during PTME.

Substrate	N	mean	median	15th percentile	75th percentile	Standard Deviation	p value for Kruskal -Wallis test	p value for Wilcoxon test
carbonate	69	–3.8	–3.8	–3.0	–4.7	1.0	<0.001	
bulk OM	29	–5.2	–4.8	–3.6	–6.1	2.0		
C_3 plant	9	–5.9	–5.7	–5.2	–7.1	1.4		
all marine	69	–3.8	–3.8	–3.0	–4.7	1.0		<0.001
all terrestrial	38	–5.4	–5.3	–4.0	–6.4	1.9		

Supplementary Table 2. Summary of $pCO_{2(background)}$, $pCO_{2(peak)}$ based on stomata, palaeosol and phythan proxy during PTME.

age (Ma)	CO_2 (ppmv)	CO_2 low (ppmv)	CO_2 high (ppmv)	proxy	period
252.09	498	374	622	Stomata[15]	$pCO_{2(background)}$
252.09	420	377	463	Stomata[15]	$pCO_{2(background)}$
252.09	347	254	440	Stomata[15]	$pCO_{2(background)}$
252.09	513	429	597	Stomata[15]	$pCO_{2(background)}$
252.09	479	406	552	Stomata[15]	$pCO_{2(background)}$
252.09	377	320	434	Stomata[15]	$pCO_{2(background)}$
252.09	421	339	503	Stomata[15]	$pCO_{2(background)}$
252.09	433	357	509	Stomata[15]	$pCO_{2(background)}$
252.09	300	272	328	Stomata[15]	$pCO_{2(background)}$
252.09	311	261	361	Stomata[15]	$pCO_{2(background)}$
252.09	438	342	534	Stomata[15]	$pCO_{2(background)}$
252.09	403	333	473	Stomata[15]	$pCO_{2(background)}$
252.09	519	316	907	Stomata[15]	$pCO_{2(background)}$
252.09	384	240	665	Stomata[15]	$pCO_{2(background)}$
252.09	368	233	635	Stomata[15]	$pCO_{2(background)}$
252.09	458	279	799	Stomata[15]	$pCO_{2(background)}$
252.09	520	323	897	Stomata[15]	$pCO_{2(background)}$
252.09	455	283	796	Stomata[15]	$pCO_{2(background)}$
252.07	400	200	800	Palaeosol[16]	$pCO_{2(background)}$
252.06	1325	662.5	2650	Palaeosol[17]	$pCO_{2(background)}$
252.06	1100	550	2200	Palaeosol[17]	$pCO_{2(background)}$
252.06	883	441.5	1766	Palaeosol[17]	$pCO_{2(background)}$
252.05	873			Phytane[18]	$pCO_{2(background)}$

252.05	1085			Phytane[18]	$pCO_{2(background)}$
251.9	1186			Phytane[18]	$pCO_{2(peak)}$
251.9	1056			Phytane[18]	$pCO_{2(peak)}$
251.9	1620			Phytane[18]	$pCO_{2(peak)}$
252.08	472	617	327	Stomata[19]	$pCO_{2(background)}$
252.08	472	666	278	Stomata[19]	$pCO_{2(background)}$
252.08	457	502	412	Stomata[19]	$pCO_{2(background)}$
252.08	687	1029	345	Stomata[19]	$pCO_{2(background)}$
252.08	805	1209	401	Stomata[19]	$pCO_{2(background)}$
251.89	1456	2682	230	Stomata[19]	$pCO_{2(peak)}$
251.89	1853	2165	1541	Stomata[19]	$pCO_{2(peak)}$
251.89	2109	3376	842	Stomata[19]	$pCO_{2(peak)}$
251.89	2019	3599	439	Stomata[19]	$pCO_{2(peak)}$
251.89	608	692	524	Stomata[19]	$pCO_{2(peak)}$
251.89	1212	1451	973	Stomata[19]	$pCO_{2(peak)}$

Supplementary Table 3. Summary of $pCO_{2(background)}$, $pCO_{2(peak)}$ and negative CIE mechanisms based on various climate models during PTME.

Model/ Proxy	negative CIE mechanism or carbon source	$pCO_{2(background)}$ (ppmv)	$pCO_{2(peak)}$ (ppmv)	ΔpCO_2 (ppmv)
Box model[20]	Combination of volcanic CO_2 (−6‰), thermogenic CH_4 (−65‰) and mass mortality	300	~1000	700
Box model[21]	mass mortality	850	2500	1650
Geochemical model and an energy balance model[22]	Combination of volcanic CO_2 (−5‰) and mass mortality	3080	9380	6300
Box model[23]	organic matter(−25‰)	1500	4000	2500
cGENIE[24]	organic matter (−25‰)	2800	8300	5500
	biogenic CH_4 (−40‰)	2800	5400	2600
	thermogenic CH_4 (−60‰)	2800	4300	1500
Box model[25]	/	845	5600	4755
Box model (LOSCAR)[26]	Combination of volcanic CO_2 (−5‰) and mass mortality	850	~4000	3150
Box model[27]	organic matter (−18‰)	500~800	4400	3600~3900

Supplementary Table 4. Parameters for the C_3 plant proxy used to reconstruct pCO_2

Input variable	Values	Source	SD or SE	Source
$\delta^{13}C_{carb}(t=0)$	3.00‰	Review of 10 marine sections (age >252.104 Ma)	0.57‰	calculated from loess fit
T(t=0)	25°C	calculated from loess fit	/	/
$\delta^{13}C_{CO2}(t=0)$	Constant	calculated from $\delta^{13}C_{carb}(t=0)$ and T(t=0)	Constant	error propagate from $\delta^{13}C_{carb}(t=0)$ and T(t=0) by Monte Carlo analysis
$\delta^{13}C_p(t=0)$	-24.42‰	Mean value (ref[15])	0.50‰	ref[15]
$pCO_2(t=0)$	425 ppmv	Mean value (ref[15])	68 ppmv	ref[15]
$\delta^{13}C_{carb}(t)$	Variable	loess fit of 10 marine sections (age =<252.104 Ma)	Variable	calculated from loess fit
T(t)	Variable	loess fit of SST compilation	Variable	calculated from loess fit
$\delta^{13}C_{CO2(t)}$	Variable	calculated from $\delta^{13}C_{carb}(t)$ and T(t)	Variable	error propagate from $\delta^{13}C_{carb}(t)$ and T(t) by Monte Carlo analysis
$\delta^{13}C_{p(t)}$	Variable	loess fit of four study sections	Variable	calculated from loess fit
A	28.26	ref[28]	0	ref[14]
B	0.22	ref[14]	0.028	ref[14]
C	Constant	error propagate from A and B by Monte Carlo analysis (ref[14])	Constant	error propagate from A and B by Monte Carlo analysis (ref[14])

Supplementary Table 5. Parameters for the simple carbon isotope mass balance calculation

Parameter	Value	Reference
k	0.3	ref[29]
$M_{background}$	66,000~82,000 Gt	refs[30, 31]
$\delta^{13}C_{background}$	2.2‰	Estimated based on global $\delta^{13}C_{carb}$ profiles (age >252.104 Ma)
$\delta^{13}C_{source}$	–6‰	ref[32]
	–25‰	ref[33]
	–40‰	ref[34]
	–60‰	ref[34]

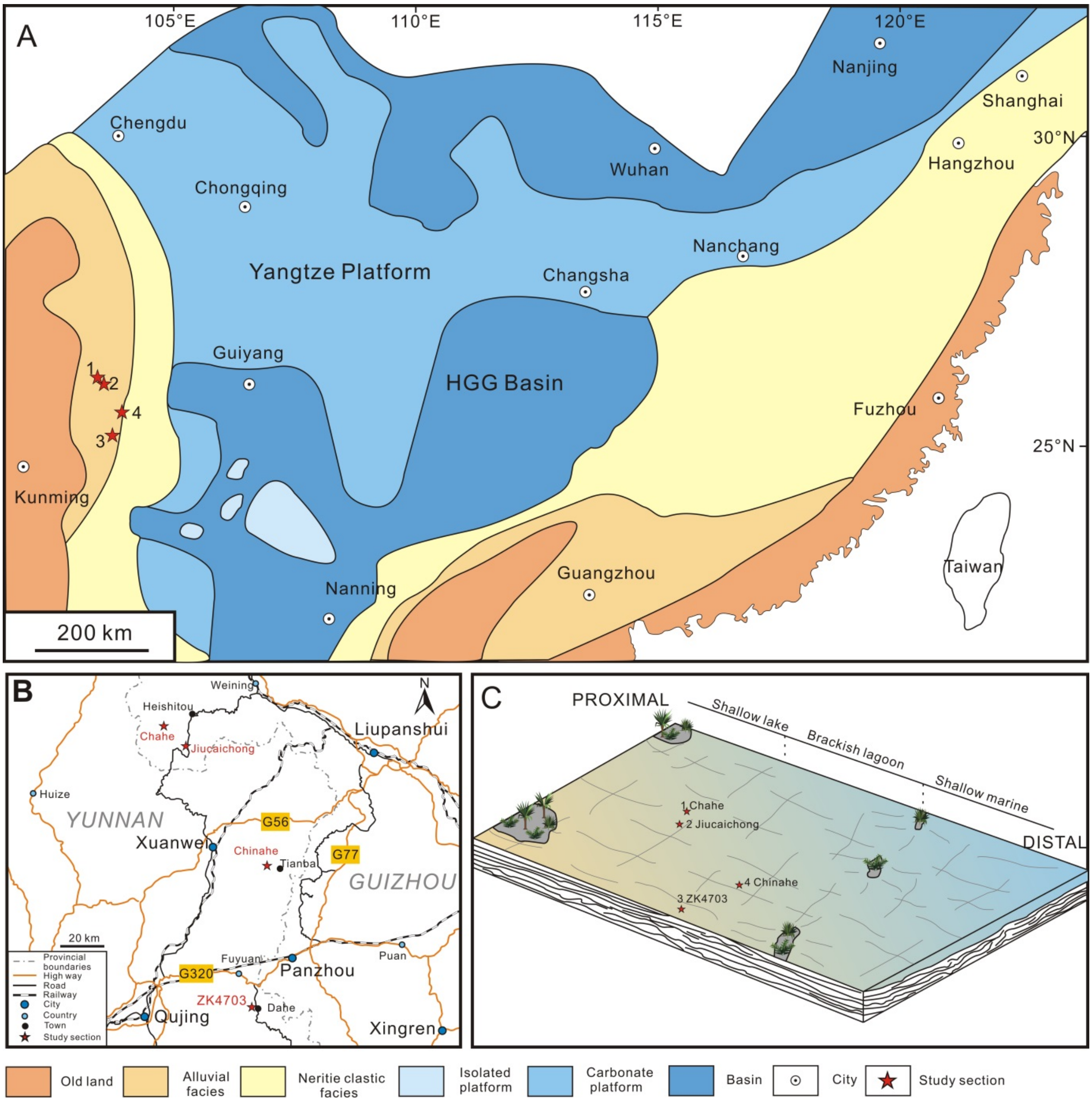

Supplementary Figure 1. (A) Palaeogeographic map of South China during Permian-Triassic boundary times (after ref[8]) showing the location of the five study sections. (B) Locations of Chahe, Jiucaichong, ZK4703 core, Chinahe and Jinzhong in western Guizhou and eastern Yunnan, southwestern China. (C) The reconstruction of the depositional environment in southwestern China during the Permian-Triassic boundary (after ref[1]), and position of the study sections.

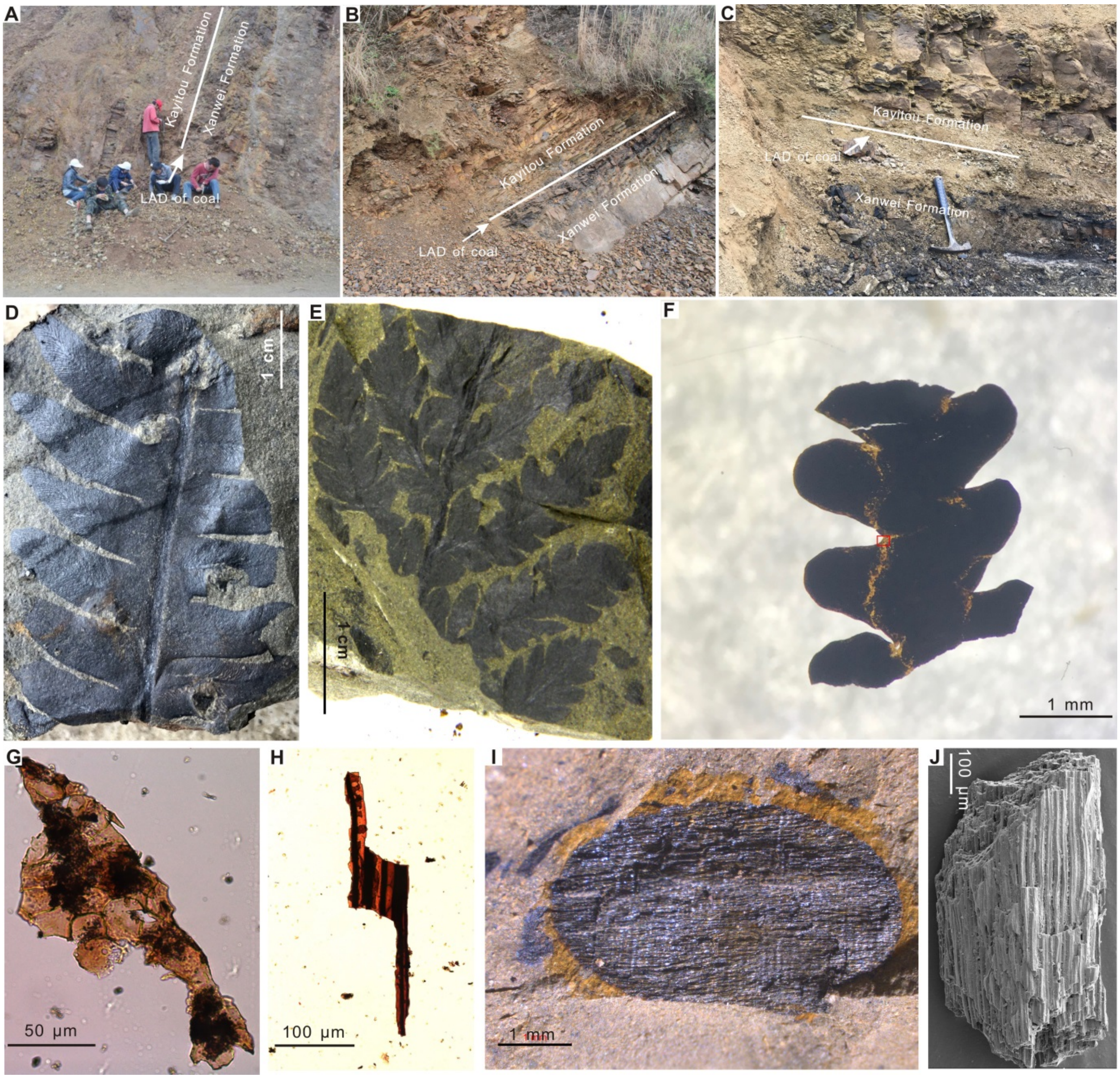

Supplementary Figure 2. Field and organic matter photos. **(A)** Lithological boundary between Xuanwei and Kayitou formations at Chahe. **(B)** Lithological boundary between Xuanwei and Kayitou formations at Chinahe. **(C)** Lithological boundary between Xuanwei and Kayitou formations at Jiucaichong. (D) Plant fossil *Compsopteris punctinervis* from Chinahe. (E) *Cladophlebis* sp. from Chinahe. (F, G) Cuticle particles under binocular microscope from ZK4703. (H) Wood particles under binocular microscope from ZK4703 . (I) Macrocharcoal particle from Chahe. (J) SEM image of microcharcoal particle from Chinahe.

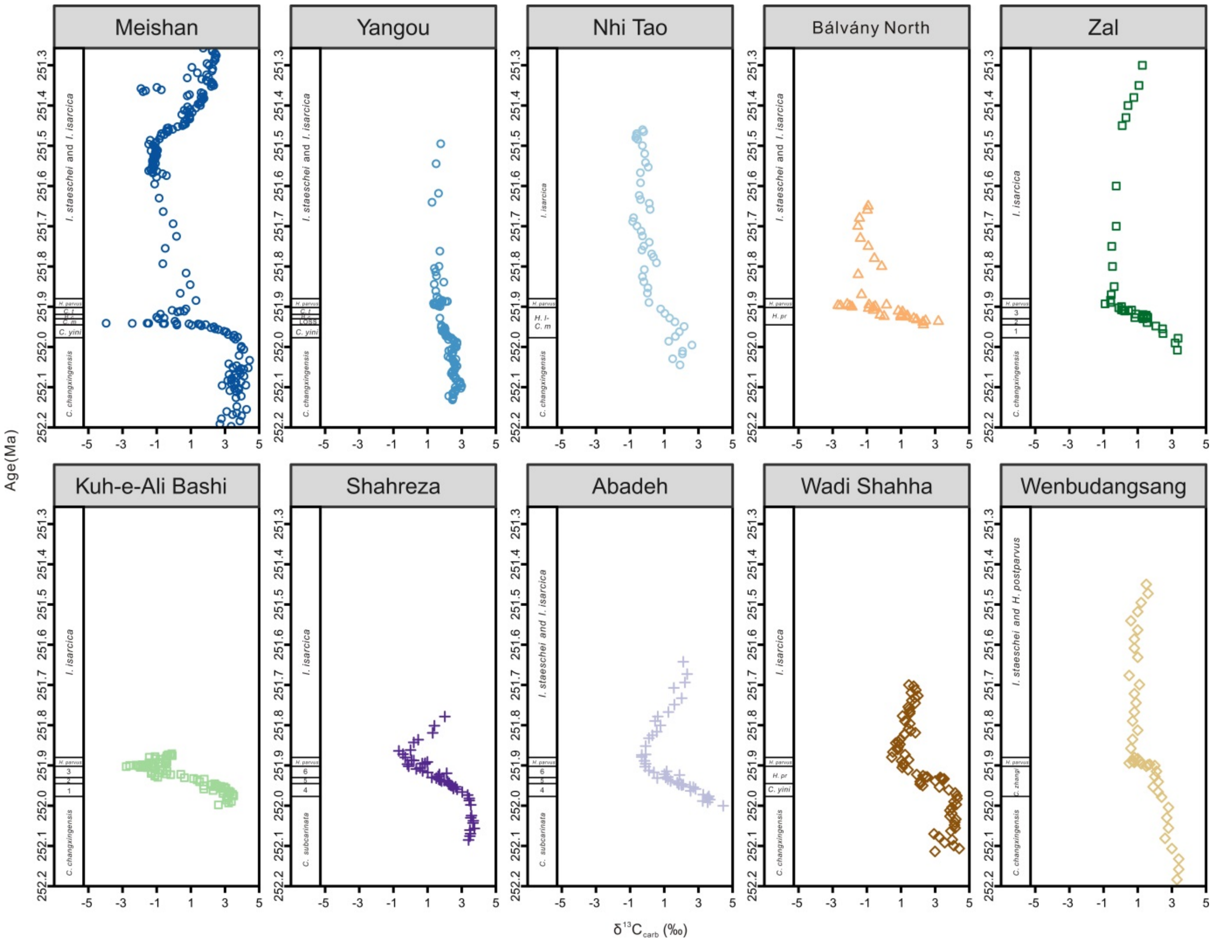

Supplementary Figure 3. The CIE shapes characterized by four stages are recorded in global marine carbonates from ten marine sections with high-resolution fata and detailed conodont zones. The conodont zones and carbon isotopes data are listed in supplementary data 2.

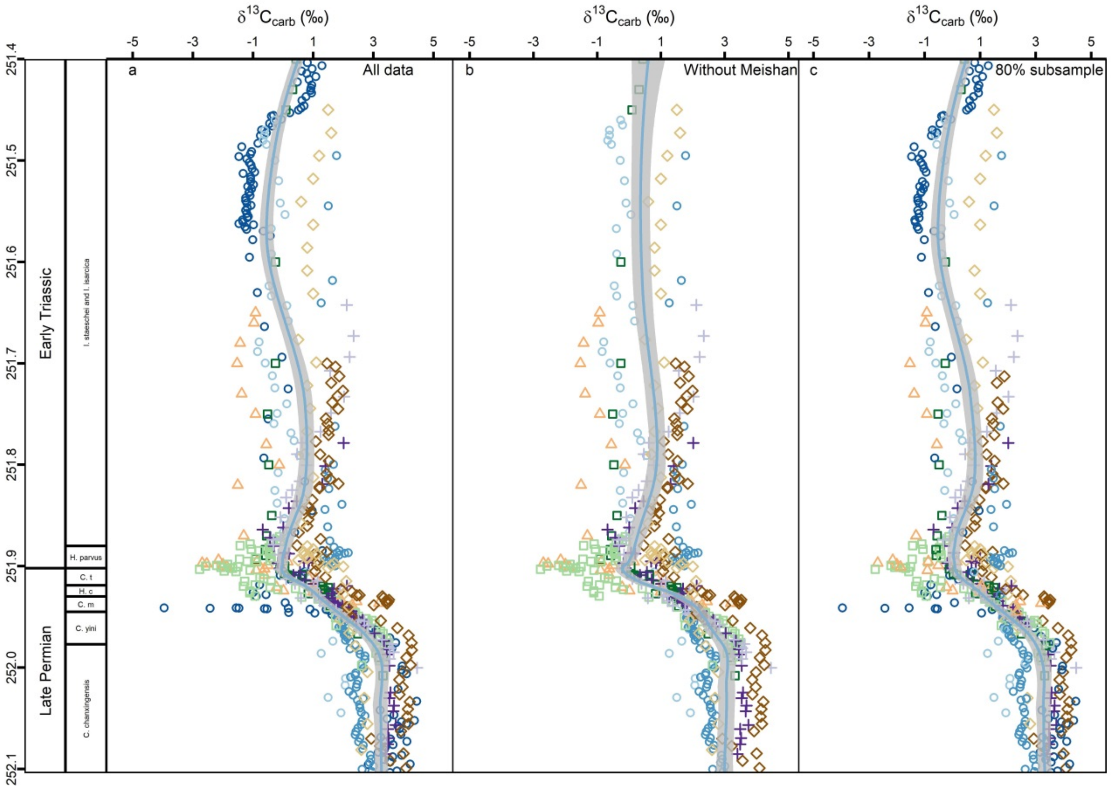

Supplementary Figure 4. a, LOESS fitting through the marine $\delta^{13}C_{carb}$ based on all data from 10 marine sections (n = 707). **b,** The LOESS fitting through the marine $\delta^{13}C_{carb}$ excluding Meishan section data (n = 508). **c,** The LOESS fitting through the marine $\delta^{13}C_{carb}$ based on 80% random subsample (n = 565).

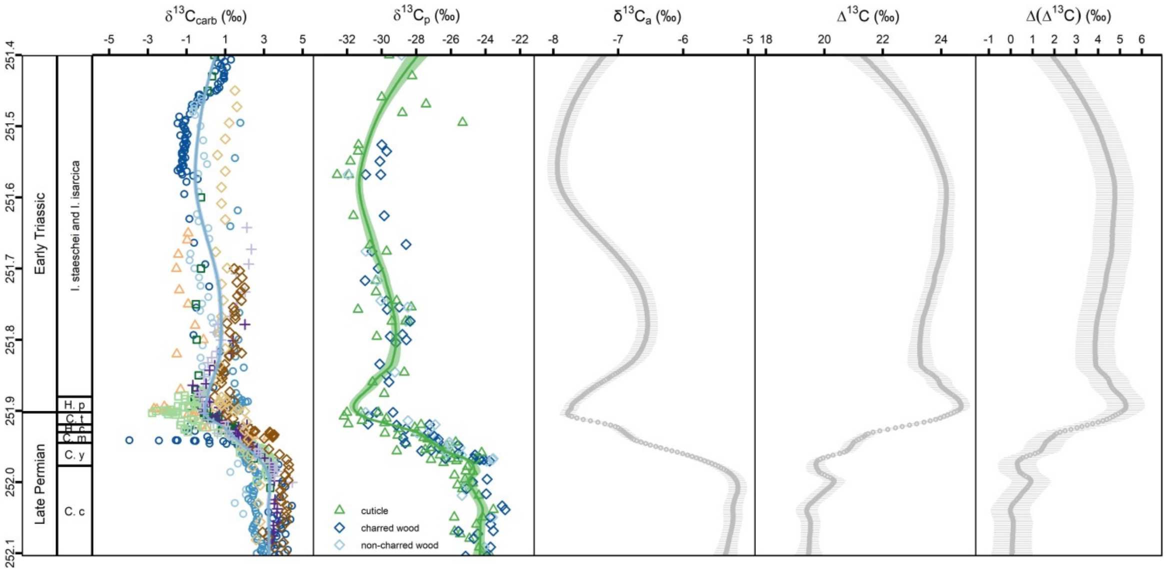

Supplementary Figure 5. The results of carbon isotope change in atmospheric CO_2 ($\delta^{13}C_a$), carbon isotope fractionation in C_3 plants ($\Delta^{13}C$) and relative change in the $\Delta^{13}C$ value between some time *t* and a reference time (t = 0) (Δ ($\Delta^{13}C$)) during the Permian-Triassic boundary interval.

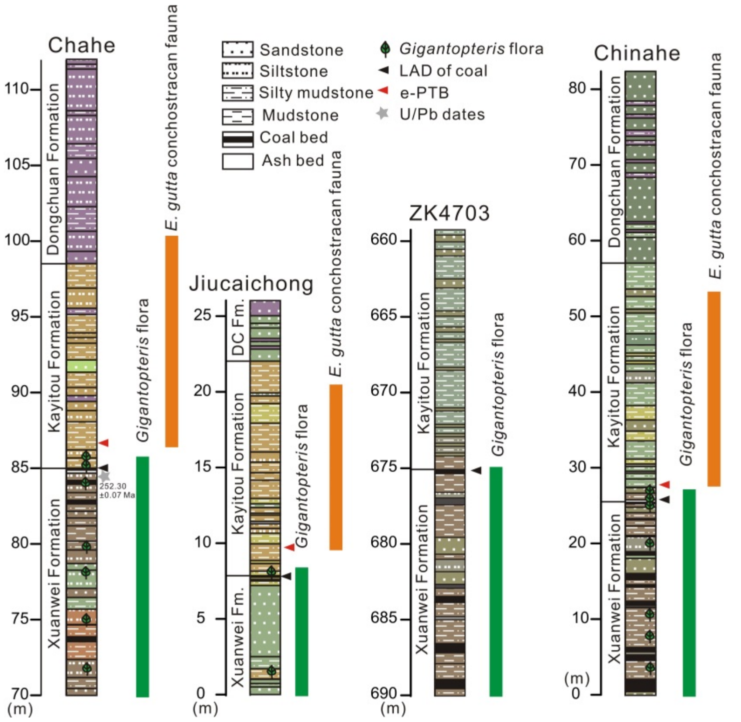

Supplementary Figure 6. Lithostratigraphy and fossil distribution in the four study section from southwestern China. The late Permian *Gigantopeteris* floral dominating the Xuanwei Formation and the base of Kayitou Formation is observed from all sections (this study; refs[1,3]). *Euestheria gutta* conchostracans fauna are discovered in Kayitou Formation and lower part of Dongchuan Formation (this study; ref[1]), whose first appearance is suggested as terrestrial estimated-PTB[1]. A U-Pb dating from top of Xuanwei Formation of Chahe section is 252.30 ± 0.07 Ma[5].

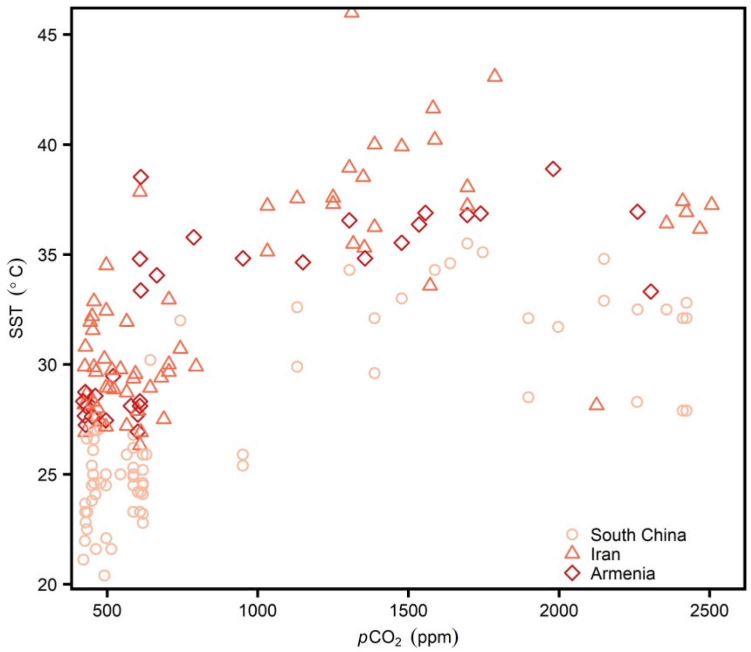

Supplementary Figure 7. The reconstructed pCO$_2$ vs. sea a compilation of surface temperature (SST) from South China[35, 36, 37], Iran[38] and Armeria[39]. The cross-plots show positive correlation between pCO$_2$ and SST (r = 0.60, p <0.001, n = 173)

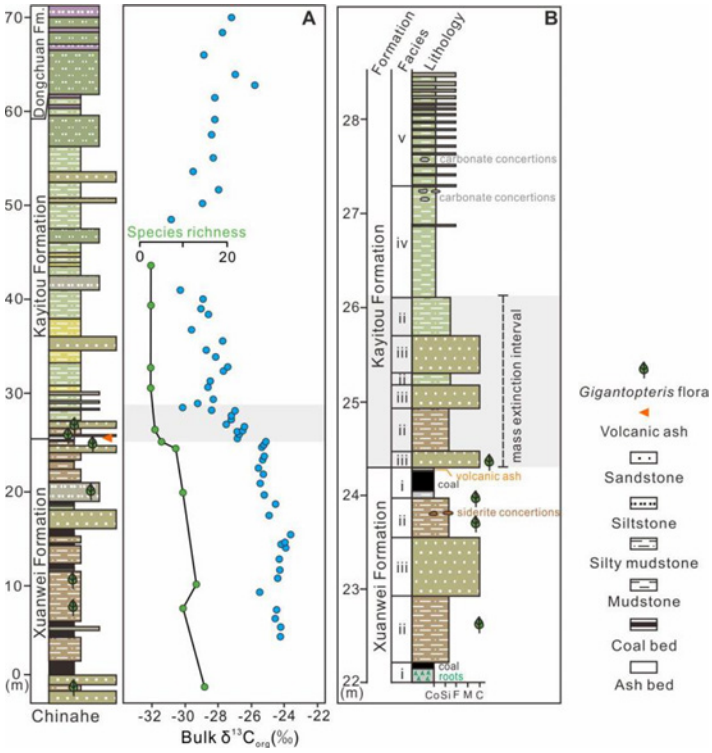

Supplementary Figure 8. Stratigraphy, plant fossil species diversity, organic carbon isotopes and detailed sedimentary log of the Permian–Triassic boundary strata at the Chinahe section. A zoom of the Permian-Triassic boundary interval at Chinahe with facies subdivision is shown in panel B

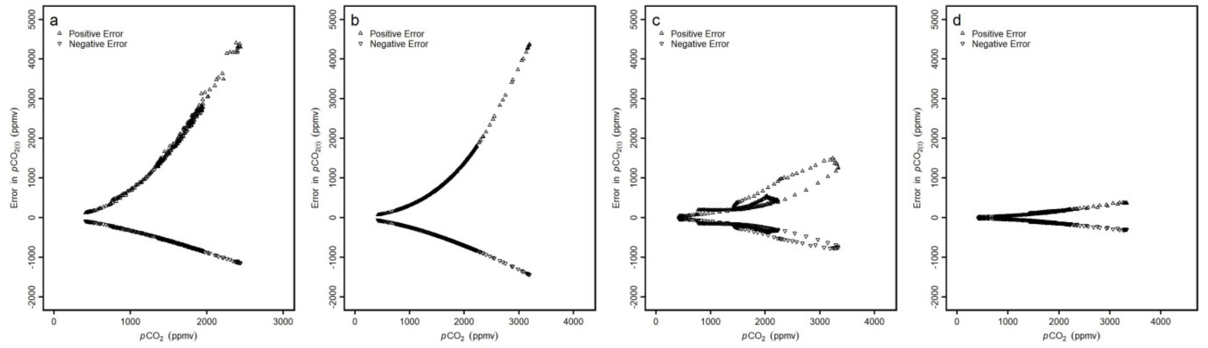

Supplementary Figure 9. a, Full error propagation showing the effect of all the uncertainties for the input listed in Supplementary Table 4 on the error in $pCO_{2(t)}$. **b**, Sensitivity analysis showing the effect of uncertainty in pCO_2(t=0) on the errors in $pCO_{2(t)}$ with all other input uncertainties set at 0. **c**, Sensitivity analysis showing the effect of uncertainty in $\delta^{13}C_p$ on the errors in $pCO_{2(t)}$ with all other input uncertainties set at 0. **d**, Sensitivity analysis showing the effect of uncertainty in $\delta^{13}C_{carb}$ on the errors in $pCO_{2(t)}$ with all other input uncertainties set at 0.

Supplementary References

1. Chu, D., et al. Biostratigraphic correlation and mass extinction during the Permian-Triassic transition in terrestrial-marine siliciclastic settings of South China. *Global Planet. Change* 146, 67–88 (2016).

2. Wignall, P. B., et al. Death in the shallows: the record of Permo-Triassic mass extinction in paralic settings, southwest China. *Global Planet. Change* 189, 103176 (2020).

3. Chu, D., et al. Ecological disturbance in tropical peatlands prior to marine Permian-Triassic mass extinction. *Geology* 48, 288–292 (2020).

4. Song, T., Tong, J., Tian, L., Chu, D. & Huang, Y. Taxonomic and ecological variations of Permian-Triassic transitional bivalve communities from the littoral clastic facies in southwestern China. *Palaeogeography, Palaeoclimatology, Palaeoecology* 519, 108–123 (2019).

5. Shen, S. Z., et al. Calibrating the End-Permian Mass Extinction. *Science* 334, 1367–1372 (2011).

6. Zhang, H., et al. The terrestrial end-Permian mass extinction in South China. *Palaeogeography, Palaeoclimatology, Palaeoecology* 448, 108–124 (2016).

7. Burgess, S. D., Bowring, S. & Shen, S. High-precision timeline for Earth's most severe extinction. *Proceedings of the National Academy of Sciences* 111, 3316–3321 (2014).

8. Yin, H., et al. The end-Permian regression in South China and its implication on mass extinction. *Earth-Sci. Rev.* 137, 19–33 (2014).

9. Yuan, D., et al. Revised conodont-based integrated high-resolution timescale for the Changhsingian Stage and end-Permian extinction interval at the Meishan sections, South China. *Lithos* 204, 220–245 (2014).

10. Korte, C. & Kozur, H. W. Carbon-isotope stratigraphy across the Permian-Triassic boundary: a review. *J. Asian Earth Sci.* 39, 215–235 (2010).

11. Shen, S. & Mei, S. Lopingian (Late Permian) high-resolution conodont biostratigraphy in Iran with comparison to South China zonation. *Geol. J.* 45, 135–161 (2010).

12. Ghaderi, A., Leda, L., Schobben, M., Korn, D. & Ashouri, A. R. High-resolution stratigraphy of the Changhsingian (Late Permian) successions of NW Iran and the Transcaucasus based on lithological features, conodonts and ammonoids. *Fossil Record* 17, 41–57 (2014).

13. Schobben, M., et al. Latest Permian carbonate carbon isotope variability traces heterogeneous organic carbon accumulation and authigenic carbonate formation. *Clim. Past* 13, 1635–1659 (2017).

14. Cui, Y. & Schubert, B. A. Quantifying uncertainty of past pCO_2 determined from changes in C_3 plant carbon isotope fractionation. *Geochim. Cosmochim. Ac.* 172, 127–138 (2016).

15. Li, H., Yu, J., McElwain, J. C., Yiotis, C. & Chen, Z. Reconstruction of atmospheric CO_2 concentration during the late Changhsingian based on fossil conifers from the Dalong Formation in South China. *Palaeogeography, Palaeoclimatology, Palaeoecology* 519, 37–48 (2019).

16. Ekart, D. D., Cerling, T. E., Montanez, I. P. & Tabor, N. J. A 400 million year carbon isotope record of pedogenic carbonate: Implications for paleoatmospheric carbon dioxide. *Am. J. Sci.* 299, 805–827 (1999).

17. Gastaldo, R. A., Knight, C. L., Neveling, J. & Tabor, N. J. Latest Permian paleosols from Wapadsberg Pass, South Africa: Implications for Changhsingian climate. *GSA Bulletin*

126, 665 (2014).

18. Witkowski, C. R., Weijers, J. W. H., Blais, B., Schouten, S. & Sinninghe Damst E, J. S. Molecular fossils from phytoplankton reveal secular $P\text{co}_2$ trend over the Phanerozoic. *Science Advances* 4, t4556 (2018).
19. Retallack, G. J. & Conde, G. D. Deep time perspective on rising atmospheric CO_2. *Global Planet. Change*, 103177 (2020).
20. Berner, R. A. Examination of hypotheses for the Permo-Triassic boundary extinction by carbon cycle modeling. *Proceedings of the National Academy of Sciences* 99, 4172–4177 (2002).
21. Rampino, M. R. & Caldeira, K. Major perturbation of ocean chemistry and a 'Strangelove Ocean'after the end-Permian mass extinction. *Terra Nova* 17, 554–559 (2005).
22. Grard, A., François, L. M., Dessert, C., Dupré, B. & Goddéris, Y. Basaltic volcanism and mass extinction at the Permo-Triassic boundary: Environmental impact and modeling of the global carbon cycle. *Earth Planet. Sc. Lett.* 234, 207–221 (2005).
23. Payne, J. L. & Kump, L. R. Evidence for recurrent Early Triassic massive volcanism from quantitative interpretation of carbon isotope fluctuations. *Earth Planet. Sc. Lett.* 256, 264–277 (2007).
24. Cui, Y., Kump, L. R. & Ridgwell, A. Initial assessment of the carbon emission rate and climatic consequences during the end-Permian mass extinction. *Palaeogeography, Palaeoclimatology, Palaeoecology* 389, 128–136 (2013).
25. Clarkson, M. O., et al. Ocean acidification and the Permo-Triassic mass extinction. *Science* 348, 229–232 (2015).
26. Komar, N. & Zeebe, R. E. Calcium and calcium isotope changes during carbon cycle perturbations at the end-Permian. *Paleoceanography* 31, 115–130 (2016).
27. Jurikova, H., et al. Permian–Triassic mass extinction pulses driven by major marine carbon cycle perturbations. *Nat. Geosci.* 13, 745–750 (2020).
28. Schubert, B. A. & Jahren, A. H. The effect of atmospheric CO_2 concentration on carbon isotope fractionation in C_3 land plants. *Geochim. Cosmochim. Ac.* 96, 29–43 (2012).
29. Panchuk, K., Ridgwell, A. & Kump, L. R. Sedimentary response to Paleocene-Eocene

Thermal Maximum carbon release: A model-data comparison. *Geology* 36, 315–318 (2008).

30. Payne, J. L., et al. Calcium isotope constraints on the end-Permian mass extinction. *Proceedings of the National Academy of Sciences* 107, 8543–8548 (2010).
31. Ridgwell, A. & Zeebe, R. E. The role of the global carbonate cycle in the regulation and evolution of the Earth system. *Earth Planet. Sc. Lett.* 234, 299–315 (2005).
32. Gales, E., Black, B. & Elkins-Tanton, L. T. Carbonatites as a record of the carbon isotope composition of large igneous province outgassing. *Earth Planet. Sc. Lett.* 535, 116076 (2020).
33. Nordt, L., Tubbs, J. & Dworkin, S. Stable carbon isotope record of terrestrial organic materials for the last 450 Ma yr. *Earth-Sci. Rev.* 159, 103–117 (2016).
34. Retallack, G. & Jahren, A. H. Methane Release from Igneous Intrusion of Coal during Late Permian Extinction Events. *The Journal of Geology* 116, 1–20 (2008).
35. Joachimski, M. M., et al. Climate warming in the latest Permian and the Permian–Triassic mass extinction. *Geology* 40, 195–198 (2012).
36. Sun, Y., et al. Lethally hot temperatures during the Early Triassic greenhouse. *Science* 338, 366–370 (2012).
37. Chen, B., et al. Permian ice volume and palaeoclimate history: Oxygen isotope proxies revisited. *Gondwana Res.* 24, 77–89 (2013).
38. Schobben, M., Joachimski, M. M., Korn, D., Leda, L. & Korte, C. Palaeotethys seawater temperature rise and an intensified hydrological cycle following the end-Permian mass extinction. *Gondwana Res.* 26, 675–683 (2014).
39. Joachimski, M. M., Alekseev, A. S., Grigoryan, A. & Gatovsky, Y. A. Siberian Trap volcanism, global warming and the Permian-Triassic mass extinction: New insights from Armenian Permian-Triassic sections. *GSA Bulletin* 132, 427–443 (2020).
