# Supplementary material for: Six-fold increase of atmospheric pCO2 during the Permian–Triassic mass extinction
Source: Nat Commun. 2021 Apr 9;12:2137. doi: 10.1038/s41467-021-22298-7 (PMC8035180; doi:10.1038/s41467-021-22298-7)
Supplement: Supplementary file 3 — Description of Additional Supplementary Information [file 41467_2021_22298_MOESM3_ESM.docx]

**Description of Additional Supplementary Files**

File name: Supplementary Data 1

Description: Global carbon isotope compilation and estimate of CIE magnitude during the Permian and Triassic mass extinction.

File name: Supplementary Data 2

Description: Marine carbonate carbon isotopes and conodont zonal correlation of ten selected marine section.

File name: Supplementary Data 3

Description: The *p*CO_2_ estimates from C_3_ plant proxy and mass of added carbon estimated from carbon isotope mass balance calculation.

File name: Supplementary Data 4

Description: Organic carbon isotope of bulk organic matter, cuticle, charred wood, non-charred wood and TOC from Chahe, Jiucaichong, core ZK4703 and Chinahe section.
